# On the Trail of Morphological Traits: Morphometry Helps to Clarify Morphological Variation in Section *Viperella* (*Sisyrinchium*)

**DOI:** 10.3390/plants13162183

**Published:** 2024-08-07

**Authors:** Julia Gabriele Dani, Camila Dellanhese Inácio, Tatiana T. Souza-Chies

**Affiliations:** Programa de Pós-Graduação em Botânica 1, Instituto de Biociências, Porto Alegre 91501-970, Rio Grande do Sul, Brazil; camilainacio1@gmail.com

**Keywords:** Iridaceae, species complex, taxonomy

## Abstract

*Sisyrinchium*, a large genus of the Iridaceae family, is divided into ten sections and defined by genetic, morphological and phylogenetic traits. The section *Viperella*, though monophyletic, encounters taxonomic hurdles, particularly within the *Sisyrinchium palmifolium* L. and *Sisyrinchium vaginatum* Spreng complexes, resulting in numerous misidentifications. The taxonomic confusion in the group may stem from various factors, emphasizing extensive morphological variations, leading to overlapping characteristics. We used morphometric approaches to better characterize the species belonging to two complexes, assess their variation and identify diagnostic traits for taxonomy enhancement. We assessed 16 quantitative traits for the *S. palmifolium* complex and 15 for the *S. vaginatum* complex, totaling 652 specimens recorded across 15 herbaria covering the entire species’ distribution area. In the *S. vaginatum* complex, 66.5% of the variations were accounted for in the first two axes, while in the *S. palmifolium* complex, the first two axes explained 55.3%. Our findings revealed that both complexes exhibited many morphological variations, leading to a characteristic overlap. These characteristics may have arisen due to recent diversifications of the group and niche overlaps. Additionally, we identified some morphological characteristics that are useful for distinguishing species. Finally, we compiled a section gathering all useful characteristics for species delimitation within the group, aiming to facilitate non-experts in deciphering this species complex.

## 1. Introduction

*Sisyrinchium* L. is one of the largest genera in the Iridaceae family, subdivided into ten monophyletic sections [1] and presents a wide geographical distribution throughout the American continent [2]. Throughout a biogeographic study [3] for the genus, it was estimated that the origin of *Sisyrinchium* included a large geographic region encompassing the southern Andes and Patagonia, central–northern Andes and Central America and Mexico. This study also claimed that the diversification of the *Viperella* Ravenna section of *Sisyrinchium* started only approximately 3.5 Mya, probably preceded by extinction in the southern Andes and Patagonia, followed by successful establishment in the southeastern highlands. Sympatric speciation took place in this latter region followed by independent dispersal events to Pampa and Chaco and the central–northern Andes. In the most recent phylogeny, *Viperella* appeared as monophyletic with strong support [1]. However, all phylogenies reconstructed for the genus have shown a lack of resolution within the section, making it difficult to clarify relationships among species [1,4,5]. In this evolutionary scenario, when distinguishing species morphologically, some taxa do not have clear boundaries, mainly in two taxonomic complexes, namely, *S. palmifolium* and *S. vaginatum*.

**Figure 1:**
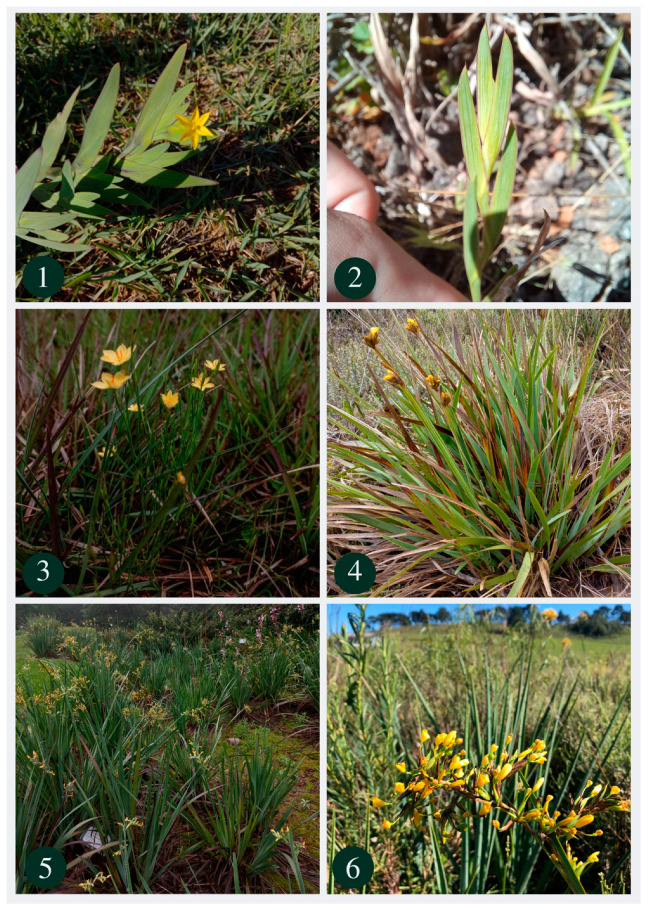
Representatives of the section *Viperella*. 1. Flower and leaves of *Sisyrinchium marchio* (Vell.) Steud. 2. Leaves of *Sisyrinchium marchioides* Ravenna. 3. *Sisyrinchium vaginatum* Spreng. 4. Leaves and inflorescence of *Sisyrinchium coalitum* Ravenna. 5. Leaves and flowers of *Sisyrinchium palmifolium* L. 6. Inflorescence and leaves (background) of *Sisyrinchium bromelioides* R. C. Foster. Source: Prepared by the authors.

In *Viperella*, we recognized two morphological groups comprising species similar to *Sisyrinchium palmifolium* L. and *S. vaginatum* Spreng., respectively (Figure 1), considered to be two distinct species complexes. Historically, these species belonged to two different sections, *Viperella* and *Hydastylus* Baker, based on vegetative characteristics. The *S. palmifolium* complex has basal leaves and only a floriferous stem and numerous spathes grouped in a congested synflorescence and belonged to *Hydastylus*. The *S. vaginatum* complex does not have basal leaves and has an erect stem with cauline leaves, which justifies its inclusion in the section *Viperella* [1]. The phylogenies performed [1,4] showed that these groups belong to the same section as they form a clade. In both morphological groups, the flowers are very similar and show little morphological variation among individuals, while vegetative traits and inflorescence organization are more informative.

Recently, *Sisyrinchium* was the object of taxonomic study, particularly the section *Viperella*, which presents several taxonomic challenges. In 2011, the first phylogeny of the genus was conducted [4], and, recently, a new phylogeny provided the most recent phylogenetic reconstruction and infrageneric classification, as well as elaborated identification keys for all sections of the genus [1]. Furthermore, a phylogenetic approach for *Viperella* was used to analyze its cytogenetic evolution in the group. Additionally, a new phylogeny was performed together with diversification analyses, but, in this case, the option of forcing monophyly was selected in the analysis [3]. Despite this, none of these trees, except that of [3], showed a clear and well-supported delimitation between the species of the two complexes.

Currently, the delimitation of the *S. vaginatum* and *S. palmifolium* species complexes is primarily supported by categorical characteristics. Despite this, there are still much confusion in the identification and differentiation of the species. The incorrect identification by non-experts could harm several studies that use these records. Frequently, plants belonging to species of the *S. palmifolium* complex are identified by non-specialists as *Sisyrinchium palmifolium*, increasing the number of occurrences of this species when, several times, the collections belong to other species of the group. Incorrect identification may also imply conservation problems, once it compromises information about rarity or conservation status [6,7]. Incorrect identifications can occur due to various factors, such as a poor taxonomic delimitation of the group, lack of diagnostic characteristics or overlapping characteristics, among others [8].

Due to the difficulty in delineating species boundaries within the two species complexes of the *Viperella* section, this study aimed to (1) understand whether the amplitude of morphological variation leads to species overlap, affecting correct identification and (2) find diagnostic morphological traits to distinguish the species. To achieve these objectives, morphometric analyses were applied. Initially, a PCA was performed to assess which characteristics explained the variation within each group.

## 2. Results

### 2.1. Principal Component Analysis (PCA)

#### 2.1.1. *S. palmifolium* Complex

**Figure 2:**
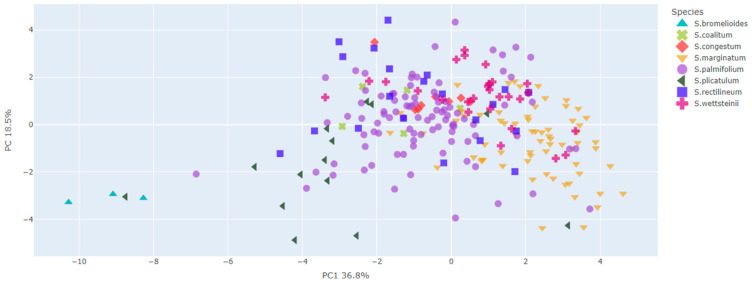
Ordination resulting from PCA based on morphometric data of the *Sisyrinchium palmifolium* L. complex. The graphic shows the distribution of the individuals in a multivariate space. Each color represents a species and the first two axes together explained 55.3% of variations.

**Figure 3:**
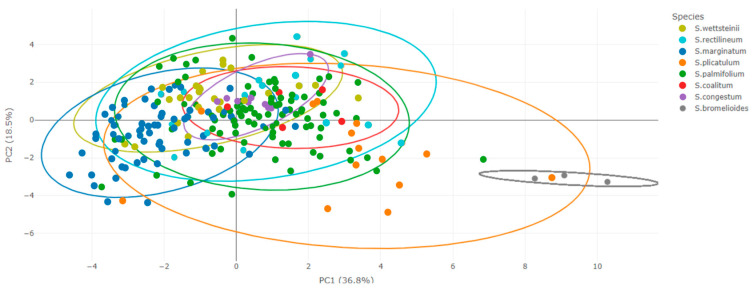
PCA with ellipses for *Sisyrinchium palmifolium* L. complex: each color represents a species. Some species, such as *Sisyrinchium bromelioides* R. C. Foster (grey), showed better delimitation compared to other species, which exhibited significant overlap.

For the *S. palmifolium* complex, the PCA explained an expressive amount of data variations corresponding to 55.3% of the variances in the first two axes (Figure 2 and Figure 3).

Table 1 presents the values of each characteristic along the first three principal axes. The majority of characteristics exhibited a similar influence on the variations within the group. Except for some characteristics, such as the number of branches of the synflorescence, number of riphidia, peduncle length and leaf length.

**Figure 4:**
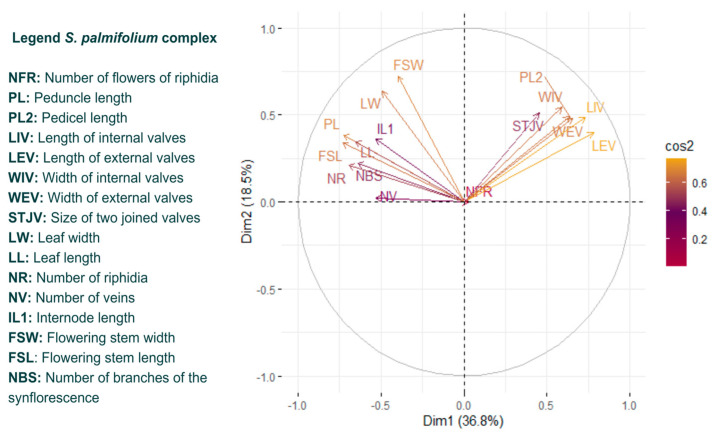
Loading plots for *Sisyrinchium palmifolium* L. complex. In yellow are the characteristics that provided the highest contribution, in purple those that provided a medium contribution and in pink those that provided the lowest contribution.

The characteristics that best explained species variations were the pedicel length, length of internal valves, length of external valves, width of internal valves and width of external valves (Figure 4).

**Figure 5:**
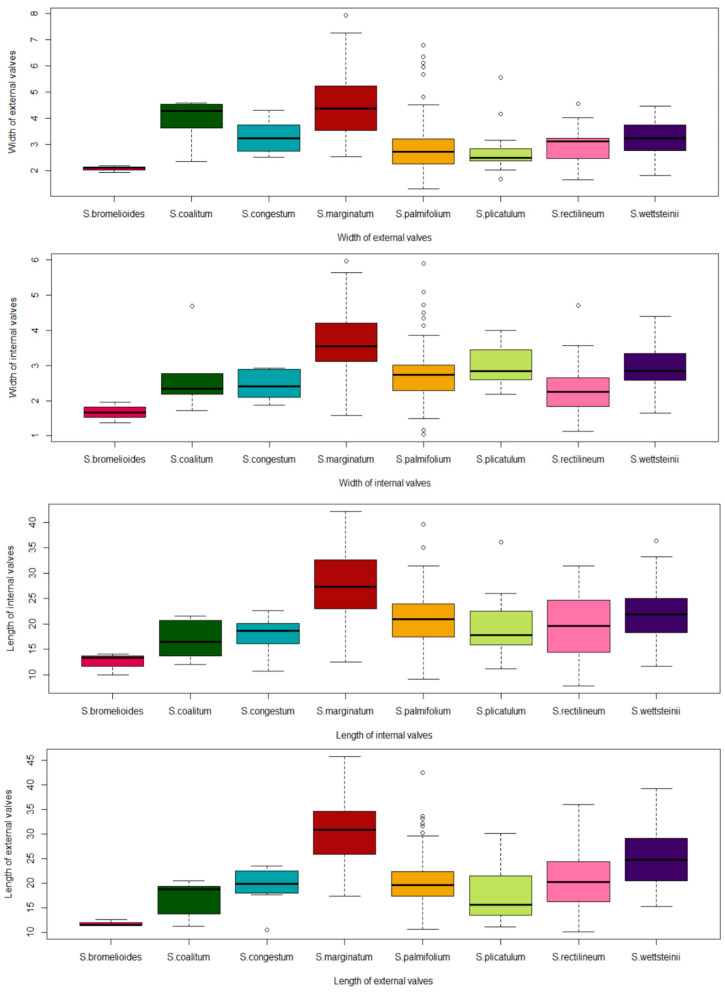
Box plots representing different characteristics that best explained the variations according to the PCA for the *Sisyrinchium palmifolium* L. complex. The black central line represents the median value for each species and the dots represent outliers. Each color represents a species.

Most of the characteristics had a similar contribution (Figure 4), except for the length of internal valves (LIV) and length of external valves (LEV). Most of the characteristics had a similar contribution, except for those that stood out and those with the lowest contribution, including the number of flowers of riphidia (NFR) and number of veins (NV). In Figure 5, we could visualize some of these characteristics.

#### 2.1.2. *S. vaginatum* Complex

**Figure 6:**
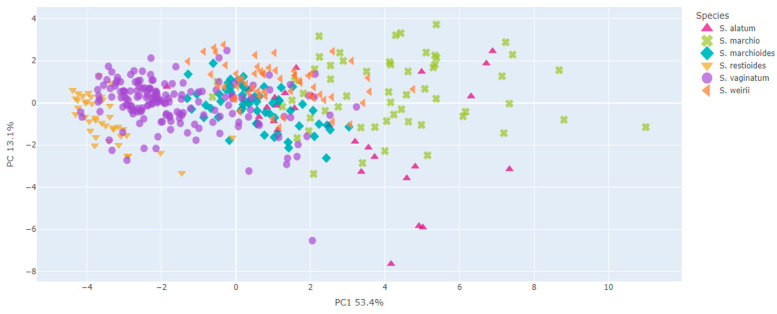
Ordination resulted from PCA of morphometric data of the *Sisyrinchium vaginatum* Spreng complex. The graphic shows the distribution of the individuals in a multivariate space. Each color represents a species and the first two axes together explained 66.5% of the variations in the group.

**Figure 7:**
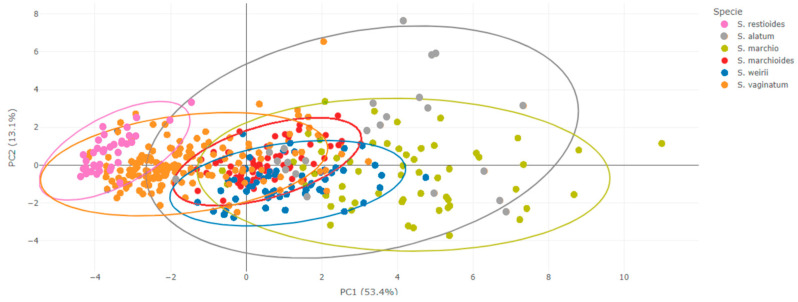
PCA with ellipses: each color represents a species. Some species, such as *Sisyrinchium restioides* Spreng (pink), showed better delimitation compared to other species, which exhibited significant overlap, particularly *Sisyrinchium vaginatum* Spreng., *Sisyrinchium marchioides* Ravenna and *Sisyrinchium weirii* Baker.

We performed a principal component analysis (Figure 6 and Figure 7) that resulted in 66.5% of the variations being explained in the first two axes for the *S. vaginatum* complex (Figure 6 and Figure 7 and Table 2).

Table 2 presents the values of each characteristic along the first three principal axes of *S. vaginatum* complexes. The majority of characteristics exhibited a similar influence on the variations within the group, except for some characteristics, including the peduncle length, length of internal valves, length of external valves, width of external valves and size of two joined valves.

**Figure 8:**
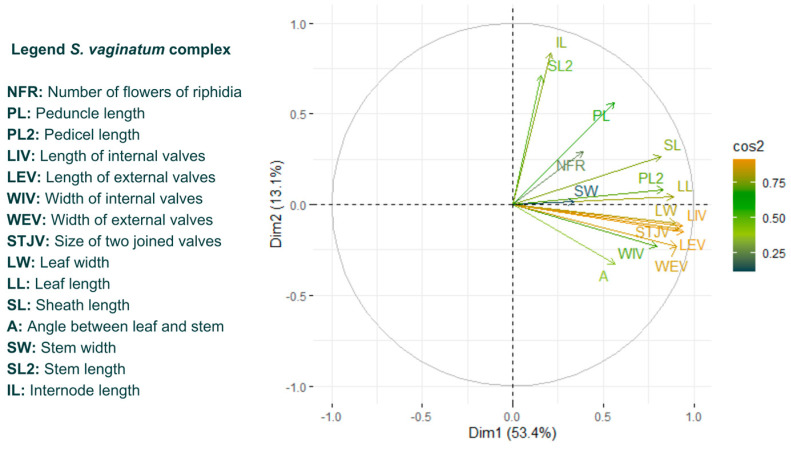
Loading plot for *Sisyrinchium palmifolium* L. complex. In yellow are the characters that provided the highest contribution, in green those that provided a medium contribution and in blue those that provided the lowest contribution.

**Figure 9:**
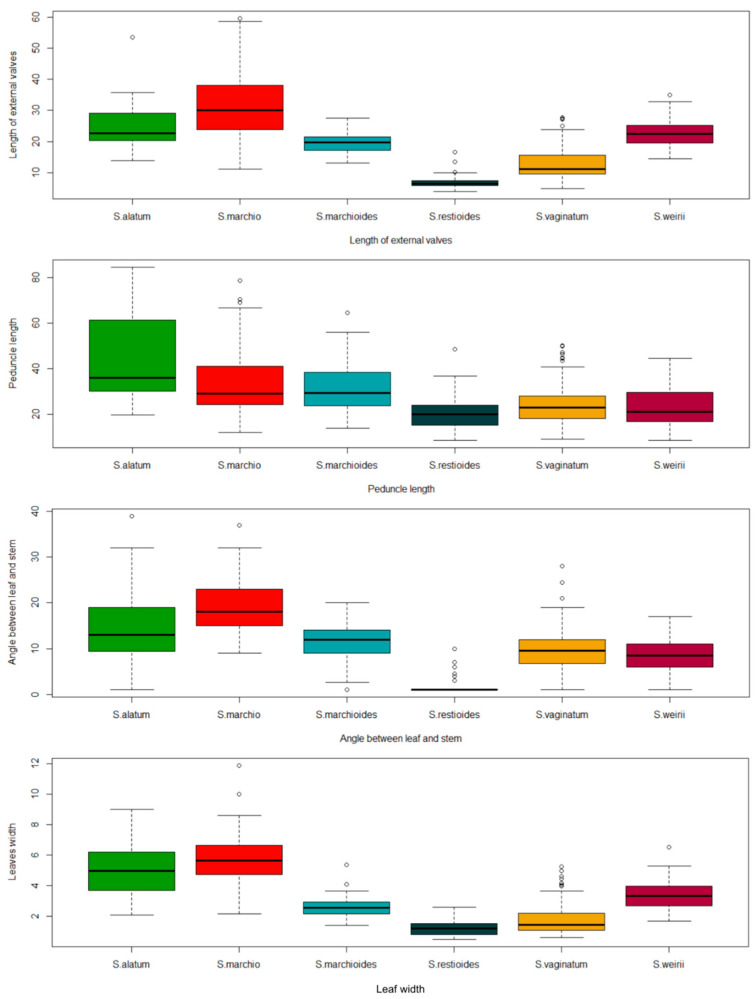
Box plots represent different characteristics that best explained the variations according to the PCA for the *Sisyrinchium vaginatum* Spreng complex. The black central line represents the median value for each species and the dots represent outliers. Each color represents a different species.

Most of the characteristics had a similar contribution (Figure 8), except for the length of internal valves (LIV), Length of external valves (LEV), Width of external valves (WEV) and size of two joined valves (STJV). Most of the characteristics had a similar contribution, except for those that stood out and those with the lowest contribution, including the number of flowers of riphidia (NFR) and number of veins (SW). In Figure 9, we could visualize some of these characteristics. Most of the characteristics had results similar to the *S. vaginatum* complex.

## 3. Discussion

### 3.1. Morphological Variations

In view of the valuable information concerning morphological variations at intra- and interspecific levels, this study involved extensive sampling to cover the entire area of occurrence of the species investigated here to better explore its morphological variations. In some cases, it was not possible to access the specimens in the field; in this case, we used only herbaria data sources. The present study included a large dataset compared to other studies based on the morphometric approach [9,10]. Additionally, we used a robust statistical basis for the data analysis to address the study questions. From our results, it was possible to verify that taxonomic confusions within the species complex mainly occurred due to the significant morphological variation among its species. As shown in Figure 2, Figure 3, Figure 6 and Figure 7, all species exhibit variations, which complicates their delimitation.

Even the characters showing greater differences between species exhibited great variations and some overlap between species (Figure 2, Figure 3, Figure 6 and Figure 7). It is possible that the recent diversification could explain the overlapping of characters found in the group [11], particularly in the PCA axes, which showed an extensive overlap in the *S. palmifolium* complex. Since the species studied here present a large intraspecific morphological variation, we observed some overlap in the PCA, making it difficult to form clearly delimited groupings. Other groups face the same problem, presenting great morphological variability, such as *Quercus* [12]. This may be related to a series of factors such as environmental variables, soil or genetic factors [13,14]. The species studied here are sympatric, meaning they co-occur geographically and share the most recent common phylogenetic history and, therefore, have experienced the same environmental influences throughout their evolutionary history [3]. These conditions could have resulted in the large characteristic overlap observed in the complexes.

### 3.2. Conclusions and Reflections on Taxonomy of S. palmifolium and S. vaginatum Complexes

Despite the presence of variations, the characteristics that best explained the variations (those related to the size of the valves) in both complexes were equivalent and were useful in delimiting the species (Figure 4, Figure 5, Figure 8 and Figure 9). Leaf characteristics are widely used in the literature to identify plants [15]. One of the advantages of using this type of trait is that it is not as ephemeral as floral characteristics, in addition to being easy to visualize in the field. For the section *Viperella*, some leaf characteristics have already been pointed out as being the most informative for the *S. palmifolium* complex [16]. In addition, some of these traits have already been used for the Flora of Brazil [17]. Here, it was possible to highlight other leaf characteristics that could also be used in the delimitation of species.

The species *S. marchio* and *S. alatum* overlapped in the PCAs. These species are difficult to delimit based on their morphology, as the measures of the morphological characteristics used here are shared by both species, both very similar morphologically. According to the results obtained here, and also due to the lack of characteristics that differentiate them, they could be the same species, since morphologically it is very difficult to differentiate them. Similar findings were supported by [17], highlighting the remarkable similarity between these species. Further taxonomic studies are currently underway to conduct more detailed investigations on these two species.

The use of more than one approach generally allows us to have a holistic view of the taxonomy of the group, integrating different lines of evidence to determine more confident taxonomic decisions [18,19]. Several studies have highlighted the importance of using more than one line of evidence in species delimitation [20,21,22]. Here, we agreed with this idea, and sought to develop a work that was more related to an integrative taxonomy. We hold the belief that authors should have a critical approach when analyzing taxonomic issues and rely on more than one piece of evidence to reach a conclusion. Through multiple investigations utilizing diverse approaches on the two complexes, species can be delineated exclusively based on morphological traits, though lacking phylogenetic evidence. We recognized the following species: *S. bromelioides*, *S. coalitum*, *S. congestum*, *S. marchioides*, *S. marginatum*, *S. palmifolium*, *S. plicatulum*, *S. rectilineum*, *S. restioides*, *S. vaginatum*, *S. weirii* and *S. wettsteinii*. According to the analyses carried out here and the lack of characteristics that differentiated the species, we did not recognize *S. marchio* and *S. alatum* as separate species. Finally, we understand that the two morphological groups, *S. vaginatum* and *S. palmifolium*, form species complexes with wide morphological variations. We acknowledge that taxa are indeed distinct species; however, due to their recent diversification and overlapping ecological niches, they exhibit very similar morphologies.

### 3.3. Conservation of Species Complexes

Conservation efforts aimed at preserving two morphological complexes studied herein are crucial for maintaining both morphological and genetic diversity. In this study, we compiled morphological data previously utilized for species delimitation and identified several important morphological characters. Phylogenetically, the species within these complexes remain not clearly separated. Nevertheless, we emphasize the importance of conserving these species complexes despite the challenges associated with their precise delimitation. Public policies directed towards such groups with ambiguous boundaries are inherently challenging, given the uncertainties in accurately estimating their geographic distributions [23,24]. However, the species within the mentioned complexes exhibited a sympatric distribution, indicating that certain regions may encompass multiple species [17]. Consequently, rather than focusing on a specific species, the objective is to preserve particular ecosystems such as the southern grasslands, which harbor numerous threatened endemic species and are largely overlooked. This approach aims to safeguard not only a single species, but the entire biodiversity associated with these ecosystems [25,26]. This strategy may be compelling for preserving the various populations occurring in these locations where there is variation in abiotic factors and, consequently, biotic factors that can significantly influence the variations within the group. Actions aimed at conserving taxa cannot wait for species complexes to be clearly defined, as it may be too late, as seen in the case of other species [27].

### 3.4. The Best Traits to Differentiate Species within the S. palmifolium and S. vaginatum Complexes

**Figure 10:**
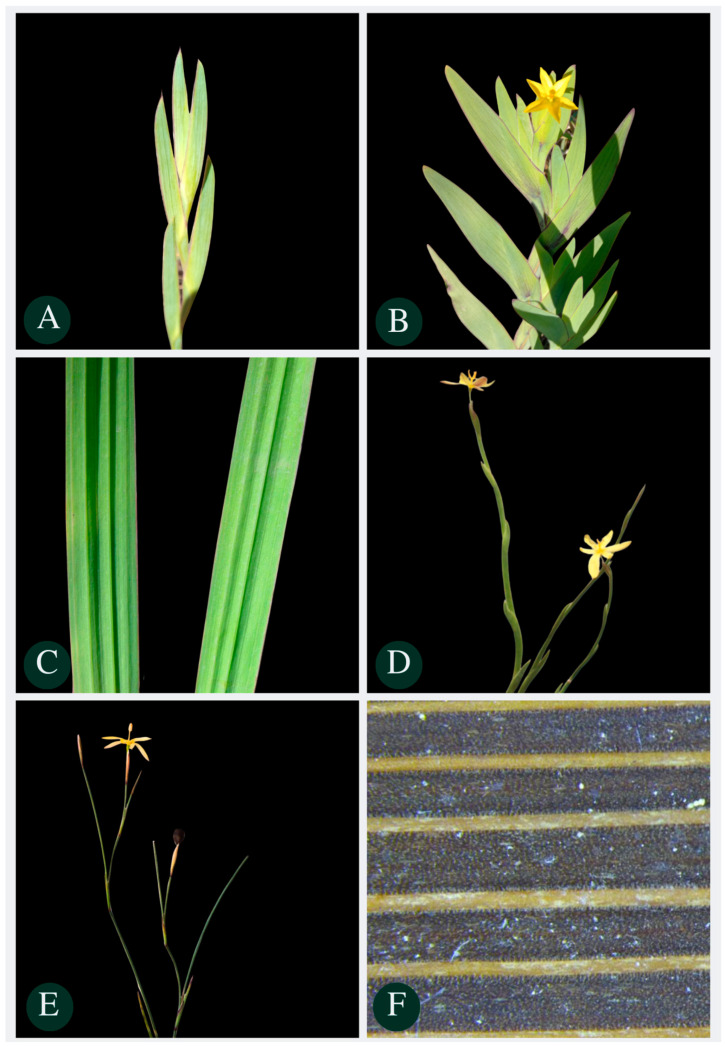
Leaves and stems of some species of the *Sisyrinchium vaginatum* Spreng complex: (**A**) leaves of *Sisyrinchium marchioides* Ravenna shortest angle in relation to the stem; (**B**) *Sisyrinchium alatum* Hook. larger leaves in relation to *S. marchioides* and angle of the leaves in relation to the larger stem; (**C**) plicated leaves of *Sisyrinchium plicatulum* Ravenna; (**D**) falciform leaves adhered to the stem of *S. vaginatum*; (**E**) linear sheets of *Sisyrinchium restioides* Spreng; (**F**) papillae on leaves of *Sisyrinchium wettsteinii* Hand.-Mazz.

**Figure 11:**
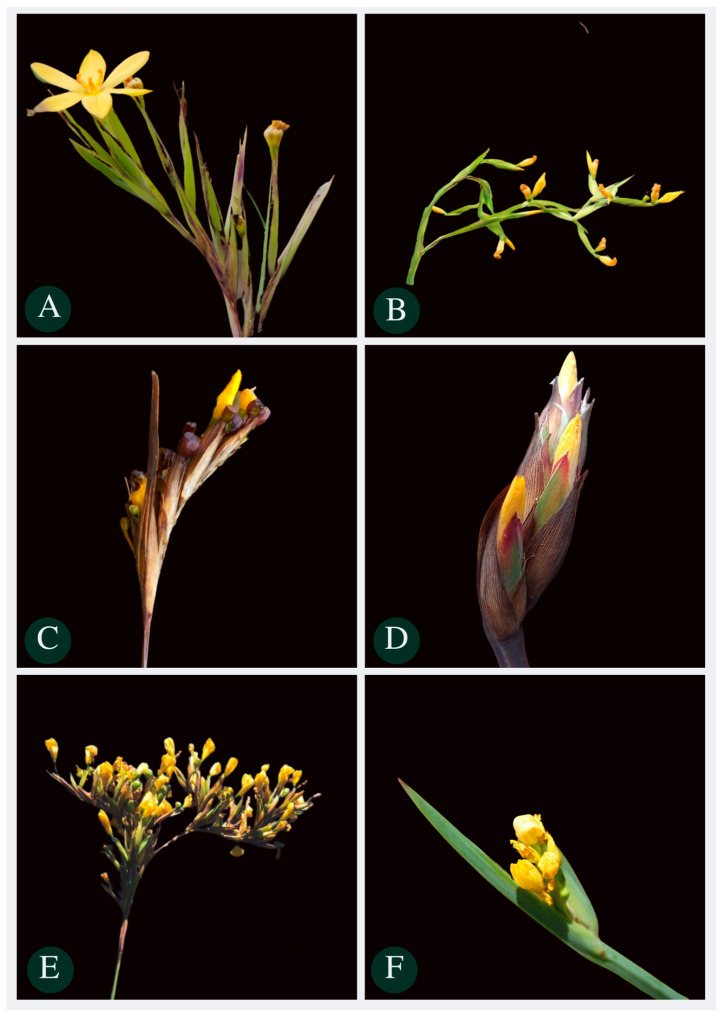
Details of inflorescence of some species of the *Sisyrinchium palmifolium* L. complex. (**A**) Pseudolateral corymbiform synflorescences of *S. palmifolium*; (**B**) pseudolateral paniculiform of *Sisyrinchium plicatulum* Ravenna; (**C**) pseudolateral congested synflorescence of *Sisyrinchium congestum* Klatt; (**D**) membranous bracteoles of *Sisyrinchium coalitum* Ravenna; (**E**) branched paniculate or elongated spiciform synflorescence of *Sisyrinchium bromelioides* R. C. Foster; (**F**) congested synflorescences of *Sisyrinchium marginatum* Klatt.

The characteristics assessed in this study, along with categorical characteristics previously employed to define the studied species, contribute to a clearer distinction among species within the complexes. The characteristics identified in this investigation are planned to be incorporated into the identification key outlined in the Flora of Brazil 2020. Additionally, we opted to integrate a concise guide comprising images (Figure 10 and Figure 11) and a table (Table 3) featuring crucial species characteristics that could prove beneficial for individuals not specialized in the field. It is imperative to emphasize that within the species studied here of the *S. vaginatum* complex, species displayed cauline leaves, whereas those within the *S. palmifolium* complex were characterized solely by basal leaves.

## 4. Materials and Methods

### 4.1. Plant Material

The *S. palmifolium* complex analyzed in the present study comprised the species *S. bromelioides* R. C. Foster, *S. coalitum* Ravenna, *S. congestum* Klatt, *S. marginatum* Klatt, *S. palmifolium*, *S. plicatulum* Ravenna, *S. rectilineum* Ravenna and *S. wettsteinii* Hand.-Mazz., and the *S. vaginatum* complex included the species *S. alatum* Hook, *S. marchio* (Vell.) Steud., *S. marchioides* Ravenna, *S. restioides* Spreng., *S. vaginatum* and *S. weirii* Baker. Here, we chose to include only those species that are taxonomically problematic, but both morphological groups include other species. Due to the great differences between both species complexes, we analyzed the groups separately. It was possible to measure 16 quantitative traits for the *S. palmifolium* complex and 15 for the *S. vaginatum* complex, 11 of them being the same in both groups (Table 4).

For this study, herbarium specimens and new collections were used. Identifications of the specimens were checked by the authors based on Flora of Brazil (the most current and comprehensive for Sisyrinchium), and three replicates for each locality were sampled. From these, five structures were measured for each specimen for each chosen trait and the final average was utilized in the analysis. Damaged material was not measured. Some species are more abundant in herbarium than others, as certain species lack collection efforts in certain localities or present limited occurrence. The same dataset was used for all statistical analyses. Materials with open flowers or fruits were always used since there are no significant changes between these two phases. Very young buds were dismissed. Regarding the angle, since they are flat leaves, when well pressed, we did not find differences between fresh and herbarium materials.

**Figure 12:**
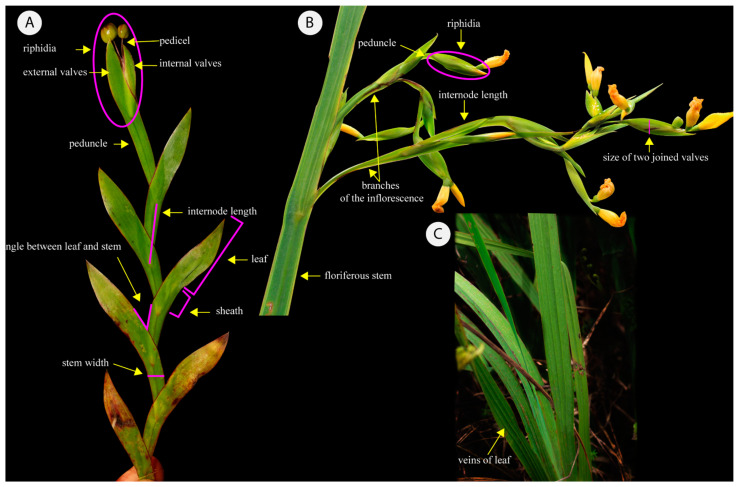
Scheme exemplifying how the measurements of morphological characteristics were obtained. (**A**) *Sisyrinchium alatum* Hook. illustrating the measurements of *Sisyrinchium vaginatum* Spreng complex; (**B**) *Sisyrinchium plicatulum* Ravenna illustrating the measurements of floral characters of *Sisyrinchium palmifolium* L. complex and (**C**) Image of the leaves indicating the leaf veins present in *S. palmifolium* complex.

The selected characteristics measured in this study are shown in Figure 12 and Table 4. When possible, the herbaria were visited, and, in some cases, digitized materials were used, including CTES, FURB, HAS, HUC, ICN, PACA-AGP, MBM, MO, MPUC, MVM, MVFA, MVJB, NY, UPCB and US (acronyms according to [28]). Furthermore, additional materials were collected in Southern Brazil (Rio Grande do Sul, Santa Catarina and Paraná) to observe the species in the field, prospecting for new populations and to search for the taxa and localities sampled. These newly collected samples were deposited in the Herbarium of the Instituto de Ciências Naturais at the Federal University of Rio Grande do Sul (ICN).

The characteristics were measured using a digital caliper; for digital materials we used the software ImageJ v. 1.53 [29]. The list with all vouchers corresponding to the measured materials is in the Supplemental Materials (Supplementary Table S1). A total of 652 specimens were used.

### 4.2. Statistical Analyses

All statistical analyses cited here were performed with R v.4.4.1 [30] with interface RStudio v.0.99.467 [31], including the descriptive analysis (box plots). Initially, we performed a principal components analysis (PCA) using data standardized to zero mean and unit variance using scale = TRUE to understand how the chosen characteristics explained the variations present in each group. To perform the calculations related to the PCA, basic functions of R were used, mainly the prcomp() that calculates the PCA. The packages used to generate the PCA graphs were Plotly [32] and ggplot2 [33]. To create the graphs, we used the function plot_ly() from the Plotly package, and to apply minor adjustments, functions like plot_bgcolor were used to adjust the background color of the graphs. Finally, we grouped the species and constructed box plots using the function boxplot() in order to verify if any selected characteristics here would be useful for separating them.

## 5. Conclusions

In the present study, we explored the morphological variation of two species complexes in *Sisyrinchium* and provided evidence of morphological characteristics that are easy to use by non-specialists. These characteristics generally included vegetative traits such as the width and length of the leaf, angle and floriferous stem width. Even those characteristics referring to inflorescence refer to persistent structures that can be found over a longer period of time, unlike floral traits, which are ephemeral and typically last a day. In the *Viperella* section, many similarities were observed.

Considering that we analyzed a recent group of species, the many interspecific similarities found in *Viperella* was normal. In addition, the great number of intraspecific variations may be linked to genetic or environmental factors. Character overlap can also be explained by niche overlapping, as the response to environmental pressures can be similar within this group. These environmental factors must be further investigated separately to help in the comprehension of which of them are more determinant in interspecific and intraspecific morphological variations. Niche modeling studies are fundamental in seeking the conservation of the group.

## Figures and Tables

**Table 1:** Morphological characters used in morphometric analyses of *Sisyrinchium palmifolium* L. complex and results of principal component analysis (PC1, PC2 and PC3).

Characteristics	PC1	PC2	PC3
Number of branches of the synflorescence	0.26393173	−0.1293314576	0.25858155
Internodes length	0.21881160	−0.2108151785	0.30092770
Number of riphidia	0.28613424	−0.1218110926	−0.18568730
Number of flowers of riphidia	−0.01125737	−0.0001886164	0.07730613
Peduncle length	0.29943317	−0.2235140373	0.13328926
Pedicel length	−0.26263855	−0.2858817375	−0.03661673
Length of internal valves	−0.30123103	−0.2827349928	−0.08139746
Length of external valves	−0.32243421	−0.2314137830	−0.23116434
Width of internal valves	−0.24307346	−0.3154324918	0.04623862
Width of external valves	−0.26986043	−0.2777715479	−0.16862665
Size of two joined valves	−0.18747736	−0.2973418950	0.01693555
Leaf width	0.20291834	−0.3700587398	0.21177190
Leaf length	0.26980714	−0.2018769742	−0.45380676
Number of veins	0.21997821	−0.0114469118	−0.52189021

**Table 2:** Morphological characteristics used in morphometric analyses of *Sisyrinchium vaginatum* Spreng complex and results of principal component analysis (PC1, PC2 and PC3).

Characteristics	PC1	PC2	PC3
Number of flowers of riphidia	0.13747231	0.20677842	−0.523204889
Peduncle length	0.19718643	0.40077302	−0.258379586
Pedicel length	0.29410032	0.05974957	−0.250559244
Length of internal valves	0.33210837	−0.08510362	−0.036316885
Length of external valves	0.33334844	−0.10676165	0.011967134
Width of internal valves	0.28139431	−0.16703078	0.002089995
Width of external valves	0.31966706	−0.16350917	0.018683360
Size of two joined valves	0.32647305	−0.09413432	−0.043390886
Leaf width	0.31895217	−0.07154315	0.140598129
Leaf length	0.31336980	0.02994528	0.271932743
Sheath length	0.28884497	0.19045932	0.261101260
Angle between leaf and stem	0.19946581	−0.23482510	0.136877383
Stem width	0.12046894	0.00995878	−0.359408787
Stem length	0.05611134	0.50647910	0.524575889

**Table 3:** Table with the best quantitative and qualitative characteristics to differentiate the most confusing species in the *Viperella* section. In the first column are the species that are most similar and in the second column are good characters to differentiate them.

Most Confusing Species	Can Be Distinguished by
*S. alatum* vs. *S. vaginatum*, *S. marchioides* and *S. weirii*	-Larger leaves in length: *S. alatum* 23.43–76.79 mm vs. *S. vaginatum* 5.3–46.18 mm; *S. marchioides* 16.04–53.85 mm and *S. weirii* 20.5–53.78 mm (rarely 57.4 mm); -Larger leaves in width: *S. alatum* 2.08–9 mm vs. *S. vaginatum* 0.59–4.96 mm; *S. marchioides* 1.4–4.07 mm (rarely 5.36 mm) and *S. weirii* 1.7–5.3 mm; -Greater angle in relation to the stem (2.5–39 °C rarely 1) (Figure 10B) vs. *S. vaginatum* (1–28 °C); *S. marchioides* (1–20 °C) and *S. weirii* (1–17 °C).
*S. bromelioides* vs. *S. palmifolium*	Differs by its robust size in *S. bromelioides*. -*S. bromelioides* length peduncle: 35–71 cm vs. *S. palmifolium* 1.9–42.93 cm; -*S. bromelioides* length floriferous stem: 150–248 vs. *S. palmifolium*, generally 16–122.5 cm (rarely 9.95 cm); -*S. bromelioides* leaf length: 114–160 cm vs. *S. palmifolium* 8.8–121; -*S. bromelioides* branched paniculate or elongated spiciform synflorescence vs. *S. palmifolium* are corimbiform pseudolateral (Figure 11E).
*S. coalitum* vs. *S. congestum*	-*S. coalitum* attenuated leaf margin vs. *S. congestum* thickened leaf margin; -*S. coalitum* glabrous leaf surface vs. *S. congestum* presence of papillae on the leaf surface; -*S. coalitum* apparent membranous bracteoles in the synflorescence (Figure 11D) vs. *S. congestum* absence of membranous bracteoles in the synflorescences and pseudolateral congested synflorescence.
*S. marginatum* vs. *S. palmifolium*	-*S. marginatum* shiny leaves vs. *S. palmifolium* matte leaves; -*S. marginatum* congested synflorescences (Figure 11F) vs. *S. palmifolium* pseudolateral corimbiforme synflorescences (Figure 11B).
*S. marchio* vs. *S. alatum*	More studies need to be conducted between *S. alatum* and *S. marchio*. Based on the current evidence, they appear to be the same species.
*S. marchioides* vs. *S. weirii*	-Leaf width: *S. marchioides* (1.4–4.07 mm (rarely 5.36 mm) vs. *S. weirii* (1.97–5.3 mm (rarely 6.52)); -External valve length: *S. marchioides* (12.98–27.58) vs. *S. weirii* (14.5–34.99 mm).
*S. plicatulum* vs. *S. palmifolium*	-*S. plicatulum* plicated leaves (Figure 10A) vs. *S. palmifolium* smooth leaves; -*S. plicatulum* winged floriferous stem which reflects on a greater sheet width vs. *S. palmifolium* non-winged floriferous stem.
*S. restioides* vs. *S. vaginatum*	-*S. restioides* nearly linear leaves attached to the stem (Figure 10E) that results in a small angle (1 °C) vs. *S. vaginatum* with a generally larger angle; -*S. restioides* leaf width (0.48–2.6 mm) vs. *S. vaginatum* leaf width (0.59–5.25 mm).
*S. vaginatum* vs. *S. alatum*, *S. restioides*, *S. marchioides* and *S. weirii*	-Leaves shaped like sickles adhered to the stem vs. *S. alatum* and *S. weirii* that have leaves with other types of shapes.
*S. weirii* vs. *S. marchioides*	-*S. weirii* presence of prominent veins on both sides of the spathe valves vs. *S. marchioides* absence of prominent veins.
*S. wettsteinii* vs. *S. marginatum*	-*S. wettsteinii* presence of papillae between leaf veins (Figure 10F) vs. *S. marginatum* absence of papillae; -*S. wettsteinii* up to 0.61 cm wide vs. *S. marginatum* 0.3–1.1 cm.

**Table 4:** Characteristics chosen for each species complex.

*S. palmifolium* Complex	*S. vaginatum* Complex
Number of flowers of riphidia	Number of flowers of riphidia
Internode length (mm)	Internode length (mm)
Pedicel length (mm)	Pedicel length (mm)
Length of internal valves (mm)	Length of internal valves (mm)
Length of external valves (mm)	Length of external valves (mm)
Width of internal valves (mm)	Width of internal valves (mm)
Width of external valves (mm)	Width of external valves (mm)
Size of two joined valves (mm)	Size of two joined valves (mm)
Leaf width (mm)	Leaf width (mm)
Leaf length (cm)	Leaf length (mm)
Peduncle length (cm)	Peduncle length (mm)
Number of veins	Angle between leaf and stem (degrees)
Floriferous stem width (mm)	Stem width (mm)
Floriferous stem length (cm)	Stem length (mm)
Number of riphidia	Sheath length (mm)
Number of branches of the inflorescence	

## Data Availability

Data are contained within the article and Supplemental Materials.

## Supplementary Materials

The following supporting information can be downloaded at: https://www.mdpi.com/article/10.3390/plants13162183/s1, Table S1: Vouchers and metrics of *S. palmifolium* complex; Table S2: Vouchers and metrics of *S. vaginatum* complex.
